# Purinergic Receptors: Key Mediators of HIV-1 Infection and Inflammation

**DOI:** 10.3389/fimmu.2015.00585

**Published:** 2015-11-26

**Authors:** Talia H. Swartz, George R. Dubyak, Benjamin K. Chen

**Affiliations:** ^1^Division of Infectious Diseases, Icahn School of Medicine at Mount Sinai, New York, NY, USA; ^2^Department of Physiology and Biophysics, Case Western Reserve University, Cleveland, OH, USA

**Keywords:** P2X, P2X7, HIV, inflammation mediators, inflammasome, inflammatory cytokines

## Abstract

Human immunodeficiency virus type 1 (HIV-1) causes a chronic infection that afflicts more than 30 million individuals worldwide. While the infection can be suppressed with potent antiretroviral therapies, individuals infected with HIV-1 have elevated levels of inflammation as indicated by increased T cell activation, soluble biomarkers, and associated morbidity and mortality. A single mechanism linking HIV-1 pathogenesis to this inflammation has yet to be identified. Purinergic receptors are known to mediate inflammation and have been shown to be required for HIV-1 infection at the level of HIV-1 membrane fusion. Here, we review the literature on the role of purinergic receptors in HIV-1 infection and associated inflammation and describe a role for these receptors as potential therapeutic targets.

## Introduction

Human immunodeficiency virus type 1 (HIV-1) disease afflicts more than 30 million individuals worldwide. The infection remains incurable despite the advent of antiretroviral therapies. Individuals who are infected with HIV-1 can live long lives without infectious complications; however, they experience non-infectious comorbidities known as non-AIDS-associated comorbidities. These are thought to be due to a process of chronic inflammation that occurs despite virologic suppression (1). This phenomenon may account for a wide variety of comorbidities including cognitive decline, cardiovascular disease, and thrombotic disease (2–8). A unifying mechanism has not been identified; however, an emerging literature implicates the role of purinergic receptors, proinflammatory signaling mediators, as important regulators of HIV-1 productive infection. Because these receptors are required for HIV-1 entry, it is hypothesized that they may additionally play a key role in inflammation and underlie comorbidities that shorten the life expectancy of HIV-infected individuals. An understanding of how these receptors may be involved in HIV-1 infection and inflammation would enable the production of novel therapeutics that both antagonize HIV-1 entry and inflammation associated with HIV-1 infection.

## HIV-1 and Inflammation

Patients with HIV-1 infection have experienced a tremendous leap in life expectancy due to the advent of effective antiretroviral therapy (ART). The result has been that individuals are living longer and now experiencing comorbidities similar to disease processes found in the general population. In fact, a study in 2008 demonstrated that only 10% of deaths in HIV-infected individuals were related to AIDS-defining illnesses while other causes included non-AIDS-defining malignancies, cardiovascular disease, liver disease, and others (9). There are certain conditions that appear to develop in HIV-infected individuals at an earlier age than the general population. This phenomenon has been referred to as “accelerated aging” and is thought to relate to chronic inflammation and immunosenescence. There are multiple possible explanations that may include ART toxicity, lifestyle (i.e., tobacco, alcohol, and IV drug abuse), as well as HIV-1 infection itself (10, 11).

How might HIV-infected individuals develop comorbidities associated with chronic inflammation? There are multiple possible explanations. HIV-1 infection causes a chronic viral infection that results in selective CD4^+^ T cell depletion which has a major impact on lymphocytes in the gastrointestinal tract (12, 13). Chronic HIV-1 infection leads to reduced integrity of the mucosal epithelium causing bacterial translocation. This process is proposed to play a key role in chronic inflammation in HIV-1 disease (14–16). High bacterial lipopolysaccharide (LPS) levels in HIV-infected individuals are associated with elevated inflammatory biomarkers (17). Abnormally high levels of T cell activation can persist despite years of virologic suppression (18), and these individuals have lower levels of CD4^+^ reconstitution (19, 20). Elevated soluble inflammatory biomarkers are detected in these individuals including markers of type I interferon (1), monocyte activation (21), and inflammation and coagulation (22). Specifically, levels of IL-6, hsCRP, and D-dimer persist at elevated levels in HIV-infected patients. There are multiple proposed mechanisms that may elevated immune activation even when virus is suppressed (1). One is that low levels of viral replication may continue, but with viral loads below the limit of detection. These levels may stimulate systemic inflammation; however, no studies support a role for intensification of therapy to reduce inflammation (23, 24). The importance of this chronic inflammation lies in its associations with comorbidities that account for the major mortality in individuals infected with HIV-1. These include cardiovascular disease, neurological decline, end organ dysfunction, and thrombotic events (2–8). Even with highly active antiretroviral therapy, which is effective at achieving virologic suppression, individuals who are chronically infected with HIV-1 have elevated inflammatory biomarkers and display innate immune activation and immune dysfunction that does not normalize with therapy (25). No unifying mechanism thus far has connected HIV-1 infection to the regulation of proinflammatory signaling.

## Overview of Purinergic Receptors

Purinergic receptors are ubiquitously expressed in mammalian cells. In 1970s, extracellular nucleotide became recognized as an important mediator of cellular signaling (26). A large literature describes the role of purinergic receptors as detectors of extracellular adenosine and adenosine triphosphate (ATP) that activate intracellular signaling events (27). These receptors can be characterized into two classes, the P1 adenosine receptors and P2 ATP/ADP receptors. P2 receptors are further divided into two categories: the P2X and P2Y subtypes. P2X receptors are ATP-gated plasma membrane channels that can be formed by a trimeric assembly of seven different subunits (P2X1–P2X7) which assemble as homotrimers or heterotrimers (28, 29).

P2X receptors are key regulators of a number of important physiological processes including neuronal synaptic and modulation, cell death and proliferation, cell and organ motility, and infection and inflammation (30–34). P2X receptors are ATP-gated non-selective cation channels. An agonist, ATP or other nucleotides, binds to the extracellular portion, inducing conformational changes that triggers channel opening and cation flux (28, 35). Some of these receptors can dilate to a larger pore, thus increasing permeability to large organic molecules (36–38). This occurs with prolonged exposure to agonist; other subtypes, notably P2X1, undergo fast desensitization with prolonged ATP exposure, resulting in closure of the channel (39, 40). Functional P2X receptors assemble as either homotrimers or heterotrimers, each subunit of which contains two transmembrane domains, a large extracellular loop containing 10 conserved cysteine residues and glycosylation sites, and intracellular N and C termini containing consensus phosphorylation sites (29, 31, 41–43). P2X7 specifically has a large pore that assembles as a homotrimer, by contrast to some other P2X receptors. Roles for the P2X7 subtype are well-characterized in the innate immune response and include proinflammatory cytokine activation, antigen presentation, and lymphocyte proliferation and differentiation (44–46). P2X7 receptor activation requires submillimolar ATP concentrations which are only transiently released extracellular compartments in response to acute cell death or injury (28). Sustained activation of P2X7 can result in large pore opening which enables passage of molecules up to 900 Da that to eventually induce cell death (31, 47).

P2Y receptors function widely across diverse physiological systems and have roles in clotting, hormone secretion, vasodilatation, neuromodulation, cell migration, cell proliferation and cell death, wound healing, and immune response (26, 27, 34, 37, 48–50). The P2Y subtypes are of G protein-coupled receptors. They consist of seven transmembrane domains with an extracellular N-terminus and an intracellular C-terminus (48, 51, 52). Activation of the receptor results in G protein dissociation into α and βγ subunits which activates downstream effector molecules. A large sequence diversity encodes for diverse pharmacological profiles among these receptors (53). There are eight P2Y monomer subtypes. P2Y1, P2Y2, P2Y4, and P2Y6 couple to G_q_ to activate phospholipase C and P2Y12, P2Y13, and P2Y14 couple to G_i_ to inhibit adenylyl cyclase and activate GIRK-family K^+^ channels. P2Y11 can couple to both *G*_q_ and *G*_s_ and trigger increases in intracellular Ca^2+^ and in cAMP levels.

## Purinergic Signaling in Inflammation

Purinergic receptors can be found in a wide variety of leukocyte sub-types, notably lymphocytes, monocyte/macrophages, and dendritic cells (29, 44, 54–56). They are critical mediators of the innate immune response in a variety of different disease states including rheumatoid arthritis, transplant rejection, and inflammatory bowel disease (57–60). Nucleotides are known mediators of innate immune cell function including cell migration (61, 62). Extracellular nucleotides, such as ATP, are released by metabolic stress, ischemia, hypoxia, and inflammation that leads to cell death and the further release of intracellular contents into surrounding tissue (26, 33). Release of ATP of through channels can signal through purinergic receptors to modify cellular orientation, cytoskeletal rearrangement, chemotaxis, and cell migration (63, 64). Studies have supported a role for signaling of these receptors in immune function of macrophages, neutrophils, B lymphocytes, and T lymphocytes. Thymocytes can undergo programed cell death in response to purinergic activation and nucleotides have been implicated in fate-determination during T cell development (65). Extracellular ATP bind these to purinergic receptor which can activate T cells through extracellular calcium influx, p38 MAPK activation, and IL-2 secretion (66–69). ATP can also activate γδ T cells through the P2X4 receptor, while P2X7 activation can promote differentiation of T into proinflammatory TH_17_ effector cells (45, 70). The P2X1, P2X4, and P2X7 subtypes are most highly expressed on leukocytes, and literature implicates the P2X7 subtype specifically in inflammatory signaling (71–74).

P2X7 receptors are the most highly expressed P2X receptor subtype in innate immune cells (44, 71, 75). They activate proinflammatory cytokine production (44, 76) and can trigger activation of the inflammasome. The inflammasome is a central scaffold protein complex that serves to coordinate interaction with caspase molecules which cleave precursor protein substrates into immunomodulatory products. Activation of P2X7 results in massive K^+^ efflux, and this change in ionic strength signals to the processing of procaspase-1 (77). Mature caspase-1 cleaves prointerleukin-1β (pro-IL-1β) into interleukin-1β (IL-1β) which is released into the cytoplasm (23, 30). Signaling takes place as part of a two component signal which requires an initial signal, such as a toll-like receptor activation via bacterial or viral ligands. Activation of P2X7 can serve as a second signal, inducing assembly of the inflammasome complex which activates caspase-1, with consequent cleavage of pro-IL-1β to mature secretory IL-1β (78). Inflammasome activation is also known to mediate pyroptosis, a mode of inflammatory programed cell death in myeloid and lymphoid leukocytes (79–81).

## Purinergic Receptors in HIV-1 Infection

Because purinergic receptor signaling can clearly mediate inflammatory responses, these receptors are likely to be activated in response to infections. As HIV-1 is a viral infection marked by chronic inflammation, this signaling pathway might serve as an important intersection between viral infection and chronic inflammation. Purinergic signaling is involved in several infectious processes (82, 83), including bacterial and mycobacterial [*Mycobacterium tuberculosis* (84–87) and *Chlamydia* infections (88)], protozoal infections including *Leishmania* (89, 90) and *Toxoplasma* (91, 92), and viral infections including respiratory viral infections (93, 94), hepatitis B and hepatitis delta virus (95, 96), hepatitis C virus (97, 98), *Cytomegalovirus* (99), and HIV-1 (100–105).

Adenosine receptors have been implicated in HIV pathogenesis as Nikolova et al. reported an association between CD39 expression and AIDS progression (106). CD39 is an ectoenzyme that breaks down ATP to AMP which in turn, is hydrolyzed by CD73 to generate adenosine that signals through purinergic A1/2-type receptors. T_reg_ inhibition was shown to be mitigated by CD39 downregulation with associated elevated levels of A2A receptor on T cells of infection patients. The authors also noted that T_reg_ CD39 expansion was associated with elevated immune activation and that a CD39 gene polymorphism was associated with reduced CD39 expression and a delay in the onset of AIDS.

A role for extracellular ATP signaling has been proposed in HIV-1 infection. Sorrell et al. observed that treatment with a non-selective P2X antagonist reduced neurotoxic effects of opiates with generated in the context of HIV Tat activity which suggested that P2X receptors might modulate neurotoxicity. Those authors proposed that P2X inhibitors may serve to reduce neuroinflammation and neurodegeration in neuro-AIDS in the context of opiate abuse (107). Tovar and colleagues found that ATP released from HIV-infected macrophages can reduce dendritic spine density through purinergic-dependent glutamate receptor down-modulation. They proposed that neuronal injury in HIV-infected patients may relate to purinergic signaling and ATP release from macrophages that can impact on glutamate regulation (108).

Recent studies have raised the possibility that purinergic receptors as host proteins may be directly related to HIV-1 pathogenesis. Seror et al. demonstrated that infection of human lymphocytes with HIV-1 can induce ATP release and that this event is required for infection (104). Pharmacologic inhibition of purinergic receptors reduced HIV-mediated cell death and HIV infection. Non-selective purinergic receptors antagonists inhibited CCR5 and CXCR4-tropic HIV-1 productive infection in lymphocytes and CCR5-tropic virus in dendritic cells and macrophages. This study found that the selective depletion of P2Y2 with small interfering RNA diminished the HIV-induced inflammatory response and also resulted in mildly elevated levels of P2Y2 in HIV-infected patient tissue compared with uninfected control tissue. Immunofluorescence analyses indicated that P2Y2 and the ATP-release channel pannexin-1 appeared to polarize to the virologic synapse; the latter is the interface between an infected donor cell and an uninfected target cell where cell-to-cell transfer and infection takes place (109, 110).

Hazleton et al. demonstrated a key role for purinergic receptors in HIV-1 replication in macrophages (102). Macrophages are critical to HIV-1 pathogenesis as they may represent key reservoirs and can mediate immune responses through production of proinflammatory cytokines. The authors demonstrated that selective pharmacologic inhibition of P2X1, P2X7, and P2Y1 resulted in dose-dependent inhibition of HIV-1 infection. Using a beta-lactamase fusion assay, they observed a requirement for P2X1 in HIV-1 fusion in macrophages and that activation of P2X1 results in calcium flux that enables HIV-1 entry (111). More recently, Giroud et al. described a role for P2X1 (112) that involved block age of binding of HIV-1 to the chemokine receptors CCR5 and CXCR4. The group corroborated findings that inhibition of P2X1 with an inhibitor did not interfere with attachment but did inhibit fusion downstream of CD4 binding prior to coreceptor engagement.

Swartz et al. demonstrated that non-selective P2X receptor inhibitors inhibit HIV-1 infection of CD4^+^ lymphocytes by cell-to-cell and cell-free mechanisms (105). Using a systematic pharmacologic screening approach, it was found that only antagonists of a P2X subclass of purinergic receptors mediated inhibition of HIV-1 viral membrane fusion and productive infection of T cells. Because P2X inhibitors are a major focus of current pharmaceutical development for chronic inflammation, pain, and depression (59, 113, 114), this drug class has variants that may be assessed for both HIV inhibitory and inflammation inhibitory activities.

Orellana and colleagues observed that the function of the pannexin-1 ATP-release hemichannel was transiently increased during early infection with both R5 and X4 tropic HIV-1 and that HIV-1 envelope binding to CD4 and coreceptors (both CXCR4 and CCR5) activates pannexin-1 channel opening as a feed-forward signal which can enable HIV-1 internalization in CD4^+^ T cells (103). This study highlights the pannexin-1 hemichannel and associated factors, i.e., purinergic receptors as host factors that play important roles in early stages of HIV-1 entry (115). The role of purinergic inhibitors in HIV-1 disease is currently being investigated (116).

Most recently, Graziano et al. demonstrated that extracellular ATP induced rapid release of HIV-1 particles from human monocyte-derived macrophages that was P2X7 dependent (117). They hypothesized that virion egress may be additionally regulated by P2X7 function.

**Figure 1:**
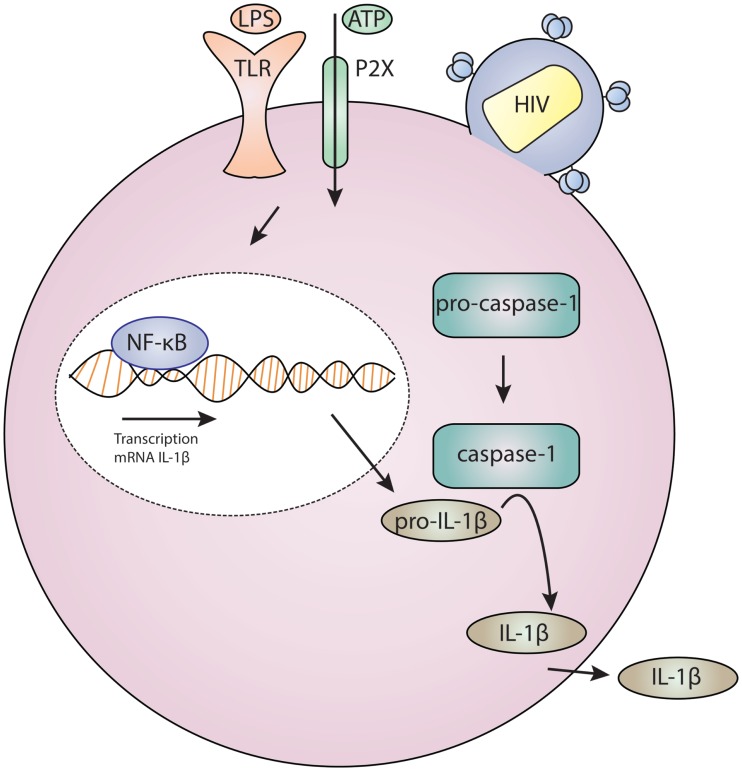
**Model for HIV infection and purinergic receptor signaling in a lymphocyte or macrophage/monocyte**. HIV-1 attaches to a cell, and this is associated with P2X activation which results in cation and potentially large molecule flux. Concurrent toll-like receptor (TLR) activation by ligands, such as bacterial lipopolysaccharide (LPS), results in gene regulation through NF-kB. These two signals – TLR and P2X – are required for inflammasome activation which results in cleavage of procaspase-1 to caspase-1 which activates IL-1β which is then secreted.

Definitive data are still lacking regarding which P2X receptor(s) are specifically required by HIV-1 and how purinergic signaling facilitates HIV-1 entry. Additionally, it is unknown whether HIV-1 infection activates other P2X7 signaling pathways, notably those involved in the NLRP3 inflammasome, which mediates IL-1β release. Elevated IL-1β is observed in HIV-infected patients (118–121), although these studies do have not directly link HIV-1 infection to inflammasome activation. Intriguing studies in CD4^+^ T cells found that pathogen sensor IFI-16 recognition of HIV-1 DNA can activate the inflammasome that induces proinflammatory lymphocyte programed cell death known as pyroptosis (122–125). This may represent a mechanism for CD4^+^ T cell depletion in HIV-1 disease and AIDS (126, 127). We present a model for the role of HIV-1 and purinergic signaling in Figure 1. This posits that HIV-1 entry results in the activation of P2X receptors and facilities fusion. This event may also trigger inflammasome activation which results in maturation and release of IL-1β; this in turn drives inflammation and inflammatory cell death, thus depleting neighboring CD4^+^ T cells and contributing to systemic inflammation.

Novel antiretroviral therapies that target both HIV-1 productive infection as well as inflammation would be helpful in treating HIV-associated comorbidities. Because targeting purinergic receptors appears to be equivalently effective at blocking cell-free and cell-to-cell infection, these are attractive targets; inhibition of cell-to-cell infection with some ART can exhibit diminished efficacy (128–130). Finally, a recent study suggests that nucleoside reverse transcriptase inhibitors can inhibit inflammasome activation and reduce levels of IL-1β production (131). This suggests an important connection between HIV-1 pathogenesis and underlying inflammation through inflammasome activation.

Well-studied purinergic compounds in advanced stages of therapeutic development are the P2X7 antagonists. Various inhibitors, such as KN-62, PPADS, oxidized ATP, brilliant Blue G, AZ9056, A 740003, and A 438079, have been tested in inflammatory and neurological diseases (132). Several highly selective P2X7 receptor antagonists have been tested in clinical trials for safety for inflammatory pain conditions, specifically rheumatoid arthritis (Table 1). All drugs tested have demonstrated safety but have yet to show efficacy at reducing inflammatory pain.

**Table 1:** **P2X7 inhibitors in clinical trials**.

Drug	Company	Phase	Endpoint	Reference
EVT 401	Evotec	I	Safety inhibition of ATP-stimulated IL-1β release	(133)
AZ9056	AstraZeneca	IIa	Safety, ACR20^a^	(58)
CE-224,535	Pfizer	IIa	Safety, ACR20	(59, 134)
GSK1482160	GlaxoSmithKline	I	Safety	(113, 135)

*^a^American College of Rheumatology 20% response criteria (136)*.

While these drugs have not demonstrated efficacy in reducing neuropathic pain, there is potential for their applications in the modulation of inflammation related to infection. Of note, suramin is a well-described antiprotozoal agent that also has reverse transcription inhibitor activity *in vitro* against HIV-1 (137). In the 1980s, suramin was proposed as an ART and was given to 98 patients with AIDS (138). The study was ineffective at demonstrating survival advantage in the treated patients, largely because the patients had a high burden of disease and because the compound is toxic. Current drug development aims for compounds with a lower molecular weight that are moderately lipophilic (132). We propose that testing these agents may yield novel classes of anti-infective drugs that can function both to reduce viral replication and associated inflammation. An important goal in this area is to clarify how purinergic receptor antagonists block HIV-1 entry and to determine the role of purinergic signaling pathways in HIV-1 pathogenesis. The development of drugs that target these pathways may aid in treatment and prevention of HIV-1 disease and associated comorbidities.

## Conclusion

Human immunodeficiency virus type 1 disease remains incurable, and as the affected population ages, patients will experience sequelae of chronic inflammation. As ART is still ineffective at eliminating this inflammation, novel therapies and a clearer understanding of the mechanisms that induce inflammation are necessary for improving long-term health of HIV-infected patients. An intriguing convergence of purinergic signaling with HIV-1 infectious pathways and inflammatory pathways indicates that these pathways may be central to disease pathogenesis. Understanding of the mechanisms that underlie such inflammation may enable targeted therapies that are more effective at enhancing the survival of HIV-infected patients by reducing chronic HIV-induced inflammation.

## Conflict of Interest Statement

The authors declare that the research was conducted in the absence of any commercial or financial relationships that could be construed as a potential conflict of interest.
